# Nonnegative Matrix Factorization: An Analytical and Interpretive Tool in Computational Biology

**DOI:** 10.1371/journal.pcbi.1000029

**Published:** 2008-07-25

**Authors:** Karthik Devarajan

**Affiliations:** Division of Population Science, Fox Chase Cancer Center, Philadelphia, Pennsylvania, United States of America; Millennium Pharmaceuticals, United States of America

## Abstract

In the last decade, advances in high-throughput technologies such as DNA microarrays have made it possible to simultaneously measure the expression levels of tens of thousands of genes and proteins. This has resulted in large amounts of biological data requiring analysis and interpretation. Nonnegative matrix factorization (NMF) was introduced as an unsupervised, parts-based learning paradigm involving the decomposition of a nonnegative matrix *V* into two nonnegative matrices, *W* and *H*, via a multiplicative updates algorithm. In the context of a *p*×*n* gene expression matrix *V* consisting of observations on *p* genes from *n* samples, each column of *W* defines a metagene, and each column of *H* represents the metagene expression pattern of the corresponding sample. NMF has been primarily applied in an unsupervised setting in image and natural language processing. More recently, it has been successfully utilized in a variety of applications in computational biology. Examples include molecular pattern discovery, class comparison and prediction, cross-platform and cross-species analysis, functional characterization of genes and biomedical informatics. In this paper, we review this method as a data analytical and interpretive tool in computational biology with an emphasis on these applications.

## Introduction

The rapid development in high-throughput technologies in the past decade has given rise to large-scale biological data in the form of expression profiles of tens of thousands of genes and proteins, often with only a handful of tissue samples. One of the objectives of a high-throughput experiment such as gene expression microarrays is molecular pattern discovery. The focus is on molecular pattern recognition via unsupervised clustering, and the identification of clusters of samples or genes revealed by their expression profiles. Analysis of genome-wide expression patterns provides unique insights into the structure of genetic networks and into biological processes not yet understood at the molecular level. Class discovery aids in the identification of hidden features in gene expression profiles that reflect molecular signatures of the tissue from which the cells originated.

Dimensionality reduction and visualization are key aspects in effectively analyzing and interpreting the high-dimensional data in this setting. Such unsupervised approaches are useful and relevant when there is no a priori knowledge of the expected gene expression patterns for a given set of genes or for any phenotype (such as experimental condition, tissue type, or patient). In studies where such prior knowledge is available, the focus is on class comparison or class prediction. In class comparison, the objective is to identify differentially expressed genes between the different classes of interest; in class prediction, however, the emphasis is on building a predictive gene set based on the class labels and expression profiles of known samples, and to apply it to a new sample to predict its class. Once a list of potentially interesting genes has been identified from these analyses, one is often interested in characterizing these genes in terms of function. In this paper, we review nonnegative matrix factorization (NMF) and its applications in computational biology, with an emphasis on the analysis and interpretation of high-throughput biological data such as those above. We discuss and illustrate the properties of NMF through examples from the literature, and provide an intuitive interpretation of the factorization and its implicit sparse nature as well as the nonnegativity constraints. In particular, we highlight its unique parts-based, local representation and contrast it with other well-known methods. In addition, we examine the usefulness of its stochastic nature in selecting an appropriate model for a given dataset and for faster implementation of the algorithm.

The paper is organized as follows. First, we introduce the basic principles underlying this method and provide a summary of its applications in computational biology. We then discuss properties unique to the NMF approach in the analysis and interpretation of large-scale biological data. Next, we address some of the limitations of this approach, and, last, we provide a discussion and some concluding remarks.

Throughout the remainder of the article, we will discuss the NMF approach in the context of class discovery (i.e., clustering samples) based on gene expression microarray experiments. This is intended only to serve as an example so as to facilitate a cogent illustration and ease of presentation of this approach. This interpretation is easily extensible to other areas of application in computational biology and should not in any way diminish the scope of the paper.

## The NMF Approach

Lee and Seung [1],[2] introduced NMF in its modern form as an unsupervised, parts-based learning paradigm in which a nonnegative matrix *V* is decomposed into two nonnegative matrices *V*∼*WH* by a multiplicative updates algorithm. They applied it for text mining and facial pattern recognition. Prior to Lee and Seung's work, a similar approach called positive matrix factorization from Paatero and Tapper [3] was applied as a dimension reduction tool to problems in the environmental sciences and astrophysics [3]–[7]. In the last few years, NMF has been widely used in a variety of areas, including image processing and facial pattern recognition [8]–[19], natural language processing such as in text mining and document clustering (see [20]–[22] and references therein), sparse coding [23]–[27], information retrieval [28],[29], speech recognition [30]–[33], video summarization [34], and Internet research [35],[36]. More recently, this approach has found its way into the domain of computational biology. We discuss its applications in this area in the next section. First, we introduce the fundamental principles underlying this approach in the context of a microarray study.

Gene expression data from a set of microarray experiments is typically presented as a matrix in which the rows correspond to expression levels of genes, the columns to samples (which may represent distinct tissues, experiments, or time points), and each entry to the expression level of a given gene in a given sample. For gene expression studies, the number of genes, *p*, is typically in the thousands; the number of samples, *n*, is typically less than 100; and the gene expression matrix, *V*, is of size *p*×*n*, whose rows contain the expression levels of *p* genes in the *n* samples.

In terms of reducing the dimensionality of the data, the objective in NMF is to find a small number of metagenes, each defined as a nonnegative linear combination of the *p* genes. This is accomplished via a decomposition of the gene expression matrix *V* into two matrices with nonnegative entries, *V*∼*WH*, where *W* has size *p*×*k*, with each of the *k* columns defining a metagene and where *H* has size *k*×*n*, with each of *n* columns representing the metagene expression pattern of the corresponding sample. The rank *k* of the factorization represents the number of latent factors in the decomposition (in our case, the number of clusters). It is generally chosen such that (*n*+*p*)*k*<*np*, i.e., a number less than *n* and *p*. Here, the entry *w_ia_* in the matrix *W* is the coefficient of gene *i* in metagene *a*, and the entry *h_aj_* in the matrix *H* is the expression level of metagene *a* in the sample *j*. It should be noted that there is a dual view of the decomposition *V*∼*WH*, which defines metasamples (rather than metagenes) and clusters the genes (rather than the samples) according to the entries of *W*.

In order to find an approximate factorization for the matrix *V*, cost functions that quantify the quality of the approximation need to be defined. Such a cost function can be constructed using some measure of distance between *V* and the product *WH*. Examples of such measures include Euclidean distance and Kullback-Leibler (KL) divergence [1],[2],[37],[38]. In the context of facial pattern recognition (and text mining) involving count data, Lee and Seung [1] derived KL divergence based on reconstruction of an image represented by *V* from *WH* by the addition of Poisson noise, i.e., *V* = *WH*+ε, where ε is a Poisson random variable.

Devarajan and Ebrahimi [39] generalized this approach based on Renyi's divergence and provided a unique framework for molecular pattern discovery using NMF. This is also based on the Poisson likelihood of generating *V* from *WH* [37]. Renyi's divergence is indexed by a parameter *α*(*α*≠1) and represents a continuum of distance measures that can be utilized for NMF based on the choice of this parameter. Various well-known distance measures arise from Renyi's divergence as special cases [37]. For example, in the limiting case *α* → 1, we obtain KL divergence given by
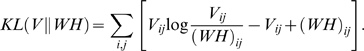
(1)This generalization unifies various competing models into a unique framework for NMF. Interestingly, Euclidean distance does not fall under this class of distance measures.

For the problem of decomposing the gene expression matrix *V* into metagenes (columns of *W*) and metagene expression patterns (columns of *H*), our goal is to minimize the objective function defined by the choice of the distance measure such as in Equation 1. Starting with random initial values for *W* and *H*, the algorithm simultaneously updates these two matrices via multiplicative rules until convergence to a local minimum is attained. Cluster membership for each sample is then determined by its highest metagene expression pattern [37],[38]. Details of the algorithm are presented elsewhere [2],[19],[23],[25],[26],[37],[38],[39]. We discuss the stochastic nature of this algorithm further in a later section.

## Applications of NMF in Computational Biology

In this section, we provide a summary of recent work on NMF with particular emphasis on applications in computational biology. While we have attempted to provide a complete and up-to-date review of its applications in a variety of problems, it is by no means comprehensive. We briefly discuss these applications here, but many of them are further discussed in detail in subsequent sections.

### 

#### Molecular Pattern Discovery

The most common application of NMF in computational biology has been in the area of molecular pattern discovery, especially for gene and protein expression microarray studies. This is an exploratory area characterized by a lack of a priori knowledge of the expected expression patterns for a given set of genes or any phenotype. However, NMF has proved to be a successful method in the elucidation of biologically meaningful classes. For instance, Kim and Tidor [40] applied NMF as a tool to cluster genes and predict functional cellular relationships in yeast using gene expression data, while Heger and Holm [41] used it for the recognition of sequence patterns among related proteins. Brunet et al. [38] applied it to cancer microarray data for the elucidation of tumor subtypes. They developed a model selection algorithm for NMF based on consensus clustering [42] that enables the choice of the appropriate number of clusters in a dataset. Similarly, Gao and Church [43] applied the Sparse NMF approach [20] for uncovering cancer subtypes using microarray data. A similar approach is described by Kim and Park [44]. Carrasco et al. [45] applied NMF for unsupervised clustering of array comparative genomic hybridization data and identified distinct genomic subtypes as well as patient subgroups in multiple myeloma (MM). Their analysis uncovered four distinct subclasses, revealing the molecular heterogeneity of MM and the division of the traditional hyperdiploid class into two subclasses.

Devarajan and Ebrahimi [37],[46] successfully applied NMF as a tool for dimensionality reduction and visualization as well as in kinetic expression profiling for analyzing microarray data (Devarajan et al., manuscript in preparation). Pascual-Montano et al. [47],[48] and Carmona-Saez et al. [49] described a method for two-way clustering of gene expression profiles using non-smooth NMF. Pascual-Montano et al. [50] also provided an analytical tool called bio-NMF for simultaneous clustering of genes and samples. For more details, the interested reader is referred to http://www.dacya.ucm.es/apascual/bioNMF/. Wang et al. [51] introduced Least Squares NMF that incorporated variability of individual measurements in microarray data. They demonstrate improved performance in terms of identification of functionally related genes based on annotations in the Munich Information Center for Protein Sequences (MIPS) database [52].

#### Class Comparison and Prediction

Recently, NMF has also been applied in a supervised learning framework such as class comparison and class prediction. Fogel et al. [53] applied this method to identify ordered sets of genes and utilize them in their sequential analysis of variance (ANOVA) procedure for identifying differentially expressed genes using microarray data. They demonstrate improved performance over traditional ANOVA in terms of power and consistency. Okun and Priisalu [54] applied it as a dimension reduction tool in conjunction with several classification methods for protein fold recognition. They show superior performance (in terms of misclassification error rate) of three classifiers based on nearest neighbor methods when applied to NMF reduced data relative to the original data. Similar applications in magnetic resonance spectroscopic imaging and fold recognition are presented in [55] and [56], respectively.

#### Cross-Platform and Cross-Species Characterization

Rapid advances in high-throughput technologies have resulted in the generation of independent large-scale biological datasets using different platforms in different laboratories. It is important to assess and interpret potential differences and similarities in these datasets in order to enable cross-platform and cross-species analyses and the eventual characterization of such data. Tamayo et al. [57] describe an approach called metagene projection for such an analysis and interpretation. Using leukemia and lung cancer data, they demonstrate that metagene projection reduces noise and technological variation while capturing invariant biological features in the data. Furthermore, they show that this approach enables the use of prior knowledge based on existing datasets in analyzing and characterizing new data [58]. In metagene projection, the dimensionality of a given dataset is reduced using NMF based on a pre-specified rank *k* factorization. An independently obtained test dataset can then be projected onto this lower, *k*-dimensional space of metagenes. This is accomplished via the Moore-Penrose generalized pseudo-inverse of *W* to obtain the projected matrix *H_p_* = *W* ^−1^ *V* (for details, see [57]). The pseudo-inverse is then applied to the test dataset and analyzed in the context of the metagenes that characterize the original data. This approach implicitly incorporates the sparse, local representation of NMF and utilizes groups of co-regulated or functionally relevant genes.

#### Biomedical Informatics

Text mining is concerned with the recognition of patterns or similarities in natural language text. The application of NMF in this area goes back to the original paper by Lee and Seung [1]. Other applications include [20],[21] and references therein. In this context, the matrix *V* is a summary of a corpus of documents in which the rows and columns represent, respectively, the words in the vocabulary and documents in the corpus. The entries of *V* denote the frequencies of words in each document. NMF is applied to identify subsets of semantic categories and to cluster the documents based on their association with these categories. Chagoyen et al. [22] present an interesting application of this approach in computational biology. Here, literature profiles are created from a corpus of documents relevant to large sets of genes and proteins using common semantic features extracted from the corpus. Genes are then represented as additive linear combinations of the semantic features, which can be further used for studying their functional associations. The authors elucidate the advantages of using NMF in identifying and interpreting the semantic features compared to other methods. Existing information about the biological entities under study can thus be used via NMF to establish putative relationships among subsets of genes and proteins that characterize a subset of the data.

#### Functional Characterization of Genes

Pehkonen et al. [59] utilize NMF for analyzing functional heterogeneity within a gene list and identifying homogeneous functional groups. In their approach, NMF is applied to the sparse, binary matrix formed on the basis of associations of relevant genes with functional classes obtained from the Gene Ontology database [60]. A non-nested hierarchical clustering scheme showing the over-represented functional groups from the gene list is created from different rank factorizations and demonstrated to better characterize groups of genes compared to current approaches. For details, please refer to [59]. This methodology is implemented in the program called GENERATOR (GENElist Aimed Theme-discovery execuTOR).

#### Other Applications

Tresch et al. [61] applied this method for the identification of muscle synergies, while Kim et al. [62] used it to determine neural activity patterns. Hiisilä et al. [63] applied this and other dimension reduction methods for assessing the dependencies between transcription factor binding sites. Other areas of applications of this method for problems involving large-scale biological data include color and vision research [64], structure-based drug design [65],[66], and magnetic resonance imaging [55],[56],[67].

## Parts-based Local Representation

There are several methods applicable for unsupervised clustering besides NMF. These include, but are not limited to, hierarchical clustering (HC), self-organizing maps (SOM), principal component analysis (PCA), vector quantization (VQ), *K*-means clustering, and multi-dimensional scaling. Hastie et al. [68] provide a comprehensive overview of these methods. Ross and Zemel [69] note that when data are represented as vectors, parts manifest themselves as subsets of the data dimensions that take on values in a coordinated fashion. While this is relevant to these methods in general, none of them have a sparse, parts-based local representation—a property that appears to be unique to NMF. Donoho and Stodden [70] provide an elegant geometric interpretation of NMF and discuss the conditions under which this approach gives a correct parts-based decomposition. In this section, we explore this particular property of NMF in detail, in the context of several applications.

### 

#### Interpretation of the Factored Matrices

The metagene coefficient *w_ia_* quantifies the influence of the *a^th^* metagene expression pattern *h_aj_* on the gene expression of the *i^th^* sample, represented by the corresponding column of the gene expression matrix *V*. For a rank *k* factorization, the relative magnitudes of the non-zero entries in each of the *k* metagenes reflect the relevance of the corresponding genes, and the expression pattern of each metagene across the *n* samples (represented by each row of *H*) reflects the relevance of the corresponding latent factor. Here, *k* is the number of clusters or hidden variables in the decomposition. The NMF framework is graphically illustrated at http://www.dacya.ucm.es/apascual/bioNMF/model.html.

The NMF representation also ensures that a single metagene expression pattern influences multiple samples. Lee and Seung [1] graphically illustrate this feature in the form of a network. In essence, the metagenes provide a summary of the behavior of genes across the samples, while the metagene expression patterns provide a summary of the behavior of samples across the genes. There is strong evidence suggesting that the metagenes and the metagene expression patterns have a sparse, parts-based representation of the gene expression data [1], [37], [38], [39], [40], [43], [46]–[50], potentially identifying local hidden variables or clusters.

NMF can be viewed as an approach for modeling the generation of gene expression measurements for samples (observable variables given by columns of *V*) from metagene expression patterns (hidden variables given by columns of *H*) [1]. In the context of clustering samples represented by the columns of *V*, the parts identify groups of samples that belong to specific clusters and are represented by the expression patterns of metagenes across samples (or the rows of *H*). In addition, genes with corresponding non-zero metagene coefficients represent groups that are co-expressed in samples. These parts provide a reduced representation of the original data, and their co-activation can be viewed as that corresponding to co-regulation or co-expression of groups of genes. Similarly, we can interpret the parts in other areas of application. For instance, in facial pattern recognition where each column of *V* corresponds to a face, the parts represent the various parts of a face such as nose, mouth, etc.; in text mining and document clustering, where each column of *V* contains word counts from documents, the parts represent the different semantic categories.

**Figure 1:**
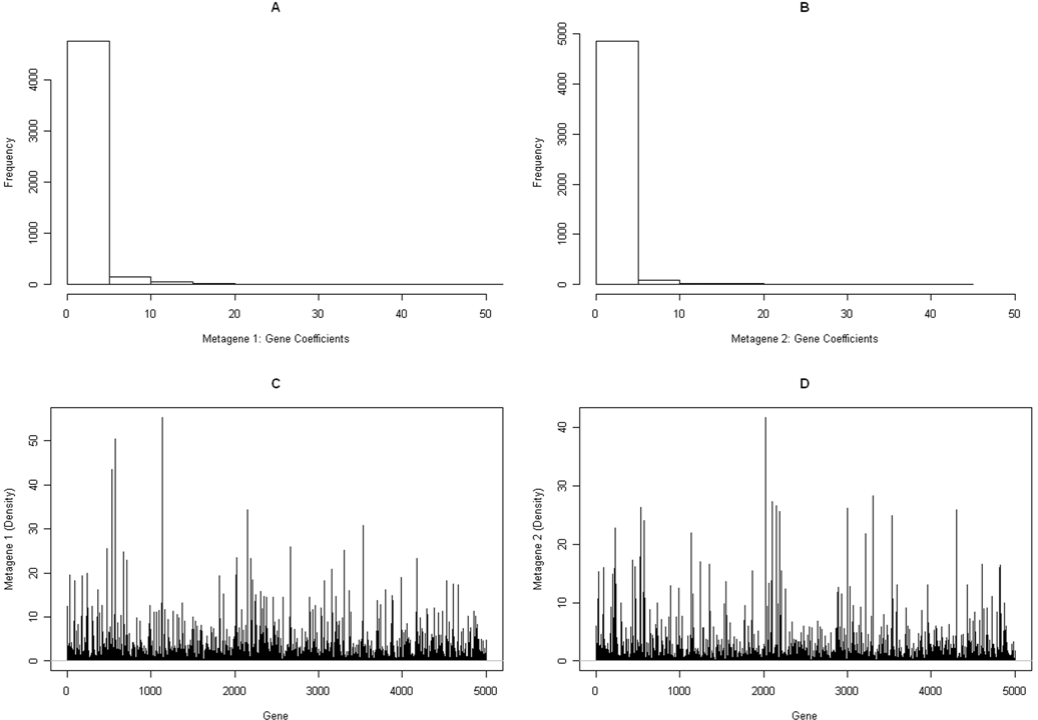
Gene coefficients. (A) Histogram of gene coefficients, metagene 1. (B) Histogram of gene coefficients, metagene 2. (C) Density of gene coefficients, metagene 1. (D) Density of gene coefficients, metagene 2.

**Figure 2:**
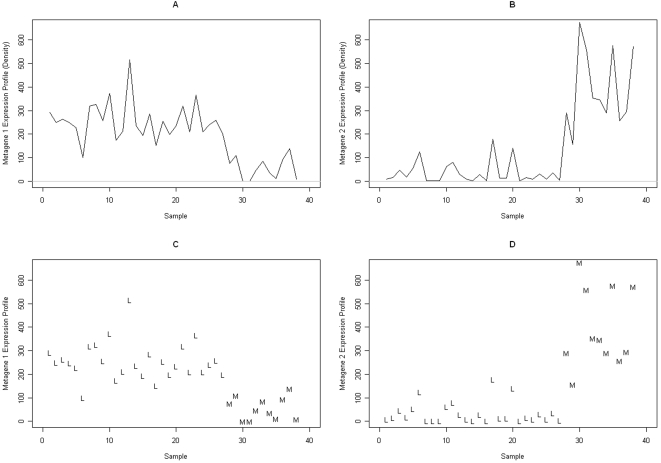
Expression profile. (A) Density of expression profile, metagene 1. (B) Density of expression profile, metagene 2. (C) Expression profile across samples, metagene 1. (D) Expression profile across samples, metagene 2.

Let us consider the widely used leukemia data available from http://www.broad.mit.edu/cgi-bin/cancer/datasets.cgi as an illustrative example. This dataset consists of 5,000 gene expression measurements each from 38 bone marrow samples from acute myelogenous leukemia (AML) and acute lymphoblastic leukemia (ALL). There are 27 ALL samples consisting of 19 B type and 8 T type, and 11 AML samples. For a rank *k* = 2 factorization, let *w*_1_ and *w*_2_ represent the two metagenes (columns of *W*) and let *h*_1_ and *h*_2_ represent the corresponding metagene expression profiles (rows of *H*). The sparseness of the metagene coefficients is illustrated in Table 1, based on a single run of the NMF algorithm using Equation 1. In this table, we list the fraction of genes whose corresponding metagene coefficients lie in the indicated range. The histograms and densities of *w*_1_ and *w*_2_ are shown in Figure 1A–1D. Only 53 and 77 genes, corresponding to *w*_1_ and *w*_2_, respectively, have coefficients that are at least 10 in magnitude. These genes may potentially behave in a strongly correlated fashion in a subset of the samples; this is determined by their metagene expression profiles across the 38 samples, *h*_1_ and *h*_2_. These expression profiles and their densities are shown in the top (Figure 2A and 2B) and bottom (Figure 2C and 2D) panels of Figure 2, respectively. Here, “L” and “M” denote, respectively, an ALL and an AML sample. It is evident from this figure that there is a clear separation between the ALL and AML samples.

**Table 1:** Distribution of metagene coefficients: Leukemia data, *k* = 2.

Coefficient	*w_1_*	*w_2_*
<1	0.730	0.840
<3	0.950	0.910
>10	0.010	0.015

#### Interpretation of Nonnegativity Constraints

The nonnegativity constraints in NMF are compatible with the intuitive notion of combining parts to form a whole, i.e., they provide a parts-based local representation of the data. This is in contrast to a holistic representation of the data provided by VQ and the distributed representation provided by PCA [1]. A parts-based model not only provides an efficient representation of the data but can potentially aid in the discovery of causal structure within it and in learning relationships between the parts [69]. In NMF, the factorization results in a reconstruction of the original data by the addition of parts due to the nonnegativity constraints, while in PCA it is a superposition of the orthogonal components with arbitrary signs that lack intuitive meaning and physical interpretation. In some applications, negative coefficients may contradict physical reality. For instance, in image reconstruction, the pixels in a greyscale image with negative intensities cannot be meaningfully interpreted.

The nonnegative coefficients also have an elegant interpretation from a neuroscience perspective. For instance, they can be interpreted as the firing rates (and synaptic strengths) of neurons in the brain, and the nonnegativity constraints account for the additive firing rates that are co-activated in physiological perception. Lee and Seung [1] propose that these constraints on firing rates may be important for developing sparse, parts-based representations for perception. The coefficients could also be interpreted as the magnitudes of muscle activation patterns that can aid in the identification of muscle synergies [61].

In the context of our gene expression theme, the nonnegative coefficients in each metagene are easily interpretable as the relative contribution of genes, unlike PCA and VQ. Returning to our leukemia example, we observe that only a small fraction of the genes (1% and 1.5%, respectively, corresponding to the two metagenes *w*_1_ and *w*_2_) significantly contribute towards the delineation of the ALL and AML samples. The identification of such a small subset of active genes is possible only due to the nonnegativity constraints which is a requirement for such a parts-based representation.

The perception of the whole is simply an additive linear combination of its parts represented in the metagenes and metagene expression profiles. Due to the nonnegativity constraints, orthogonality of metagenes and metagene expression profiles cannot be achieved in practice. However, this is an extremely useful property, since the dependence among the gene expression profiles typically present in a microarray study can be captured by overlapping metagenes. This property makes NMF particularly well-suited for the analysis of large-scale biological data, where it is essential to capture relationships underlying inter-connected biological pathways or processes. In terms of this property, NMF has been shown to be superior to other dimension reduction methods (see [20] and references therein). While the decomposition *V*∼*WH* is linear, it is important to note that the computation of the update rules for *W* and *H* is non-linear due to the nonnegativity constraints [1].

#### Enforcing Sparseness

While the original NMF approach has been shown to have a naturally sparse, parts-based, and local representation as seen in [1],[37],[38],[39],[40],[45],[46], there is also some evidence that points to a parts-based but holistic (rather than local) representation produced by NMF [19], [23]–[25]. Lee and Seung [1] note that sparseness in both the metagenes and metagene expression profiles is crucial to a parts-based representation. The nonnegativity constraints may be a necessary condition for such a parts-based representation, but they may not be sufficient to achieve sparseness. In such a case, it may be desirable to explicitly enforce sparseness on the metagenes and the metagene expression patterns. Recent work has focused on imposing such explicit sparseness constraints on the entries of *H* or *W* or both [19]–[21], [23]–[26], [43], [44], [47]–[50]. This is generally achieved via the addition of appropriate penalty terms to the objective function defined by the distance measure of our choice. For instance, one could impose a constraint on the metagene expression patterns *H*. An example of such a constraint is the sum of the entries of *H*, Σ*_aj_H_aj_*. Other penalty terms can also be used as appropriate (see [19]–[21],[25],[43]). Using KL divergence as defined in Equation 1, our objective function would then be
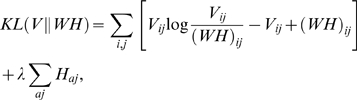
(2)where *λ*>0. The parameter *λ* quantifies the trade-off between goodness-of-fit of the model (defined by KL divergence) and sparseness.

Gao and Church applied the method outlined in [20] to cancer microarray data and explicitly enforced sparseness via the sum of squares of the entries of *H*. They demonstrated improved performance (in terms of misclassification error rate defined as the proportion of samples misclassified by the algorithm across all clusters) over standard NMF as well as identified subsets of co-expressed genes that may be involved in cancer. Pascual-Montano et al. [47],[48],[50] adopt a different approach for enforcing sparseness. They utilize a smoothing operator to simultaneously enforce sparseness on both *W* and *H*. Regardless of the approach, enforcing sparseness on the metagenes and metagene expression patterns across samples aids in the detection of sharp boundaries between different classes. We noted earlier that orthogonality of metagenes and metagene expression profiles cannot be achieved in practice due to the nonnegativity constraints. However, the enforcement of sparseness constraints decreases their overlap, thus resulting in localized, disjoint groups of samples or genes, respectively.

#### Capturing Context-Dependent Patterns

In contrast to traditional clustering and dimension reduction methods, NMF has been demonstrated to identify subtle, context-dependent biological patterns as well as being less sensitive to the selection and/or perturbation of input genes utilized in the factorization. Such context dependency is not captured by standard two-way clustering approaches [38]. For instance, NMF has been shown to be capable of identifying patterns that exist in only a subset of the samples, whereas standard methods focus on the overall structure in a dataset (i.e., on samples for which similarity in expression extends across all genes), thus overlooking the subtle features that represent relevant biological patterns [1],[38],[40]. In essence, NMF aids in the elucidation of localized patterns of similar expression by identifying a small subset of genes that act in a strongly correlated fashion in a subset of the samples. As noted before, such localized patterns may point to groups of co-regulated or functionally relevant genes [38],[43],[47],[48],[50]. For example, groups of genes and samples that show high coefficients for a given metagene (column of *W*) and the corresponding metagene expression pattern (row of *H*), respectively, may be strongly related in a subset of the data, thus constituting a gene-sample bi-cluster. Pascual-Montano et al. [47],[48] utilize this feature and have developed bioNMF, a data analytical tool for identifying gene expression bi-clusters [50].

In a study of functional cellular relationships in yeast, Kim and Tidor [40] observed that genes with relatively high coefficients in the metagenes were dominated by only a few functional categories. They showed that NMF outperformed all other methods applied, including SVD and *K*-means, in predicting functional relationships between experiments with comparison to the MIPS classification and the Yeast Proteome Database (YPD) [52]. They note that out of the 100 strongest functional relationships detected by NMF, 35 and 58 could be verified by MIPS and YPD, respectively, far exceeding those of the other methods used. Similarly, Gao and Church [43] investigated genes with high metagene coefficients corresponding to each of the three clusters, ALL-B, ALL-T, and AML, in the leukemia data described before. Among these, they identified genes that were enriched in chemokines, oncogenes, tumor suppressor genes, and DNA repair genes.

## Stochastic Nature of NMF Algorithm

NMF has proved to be an attractive method for the effective analysis and interpretation of large-scale biological data [37], [38], [39], [40], [41], [43]–[51], [53]–[57], [59], [61]–[63]. However, due to its nonnegativity constraints, it suffers from an algorithmically more complex implementation relative to a traditional clustering method like HC that is based on pairwise distance computations. There is a substantial gain in computational time due to the matrix representation of the NMF update rules. These rules guarantee convergence of the algorithm to a local minimum based on random initial values for *W* and *H*. However, the algorithm may not converge to the same solution on each run due to the stochastic nature of initial conditions, thus requiring it to be run multiple times based on random initial values for *W* and *H*. The algorithm groups the samples into *k* clusters, where *k* is the pre-specified rank of the factorization. As noted before, class membership for each sample is determined based on the highest metagene expression profile [37],[38].

### 

#### Model Selection: Choice of *k*

The stochastic nature of the algorithm has been shown to be rather useful in providing methods for evaluating the consistency and robustness of its performance. Studies have shown that 50–200 NMF runs are usually sufficient to provide stability to the clustering [37],[38]. As the number of runs increases, the metagene expression patterns across the samples become more localized with decreasing overlapping support, resulting in a sparse, localized, and compact representation [38]. This stochastic feature can be effectively utilized to assess whether a given rank *k* provides a biologically meaningful decomposition of the data.

Monti et al. [42] developed a methodology called consensus clustering for evaluating the performance of any unsupervised clustering algorithm based on resampling methods. It represents the consensus across multiple runs of the algorithm and quantifies the stability of the discovered clusters. It can also be utilized to assess the sensitivity of a stochastic method like NMF to random initial conditions. Model selection procedures that quantify the robustness of the factorization via consensus clustering have been developed and applied to NMF [37],[38]. In the case of NMF, its stochastic nature is utilized in the evaluation process, where information from each run of the algorithm is combined as outlined below.

Suppose that we are applying NMF to cluster *n* samples. For a factorization of given rank *k*, each run of the algorithm results in an *n*×*n* connectivity matrix *C* with an entry of 1 if samples *i* and *j* cluster together and 0 otherwise, where *i*,*j* = 1,…,*n*. The consensus matrix *C̅* is simply the average connectivity matrix obtained over multiple runs of the NMF algorithm. Final sample assignments and cluster visualization are based on the re-ordered consensus matrix. The robustness of each factorization is evaluated by computing the cophenetic correlation coefficient *ρ* where 0≤*ρ*≤1. A high value of *ρ* indicates homogeneous clusters. Brunet et al. [38] advocate the use of *ρ* as a single measure for choice of the appropriate number of clusters by plotting *ρ* for various choices of the number of clusters *k*.

**Figure 3:**
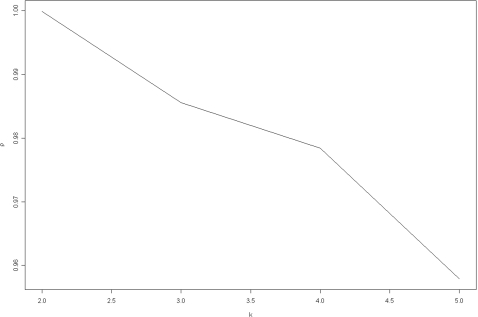
Cophenetic correlation coefficient.

**Figure 4:**
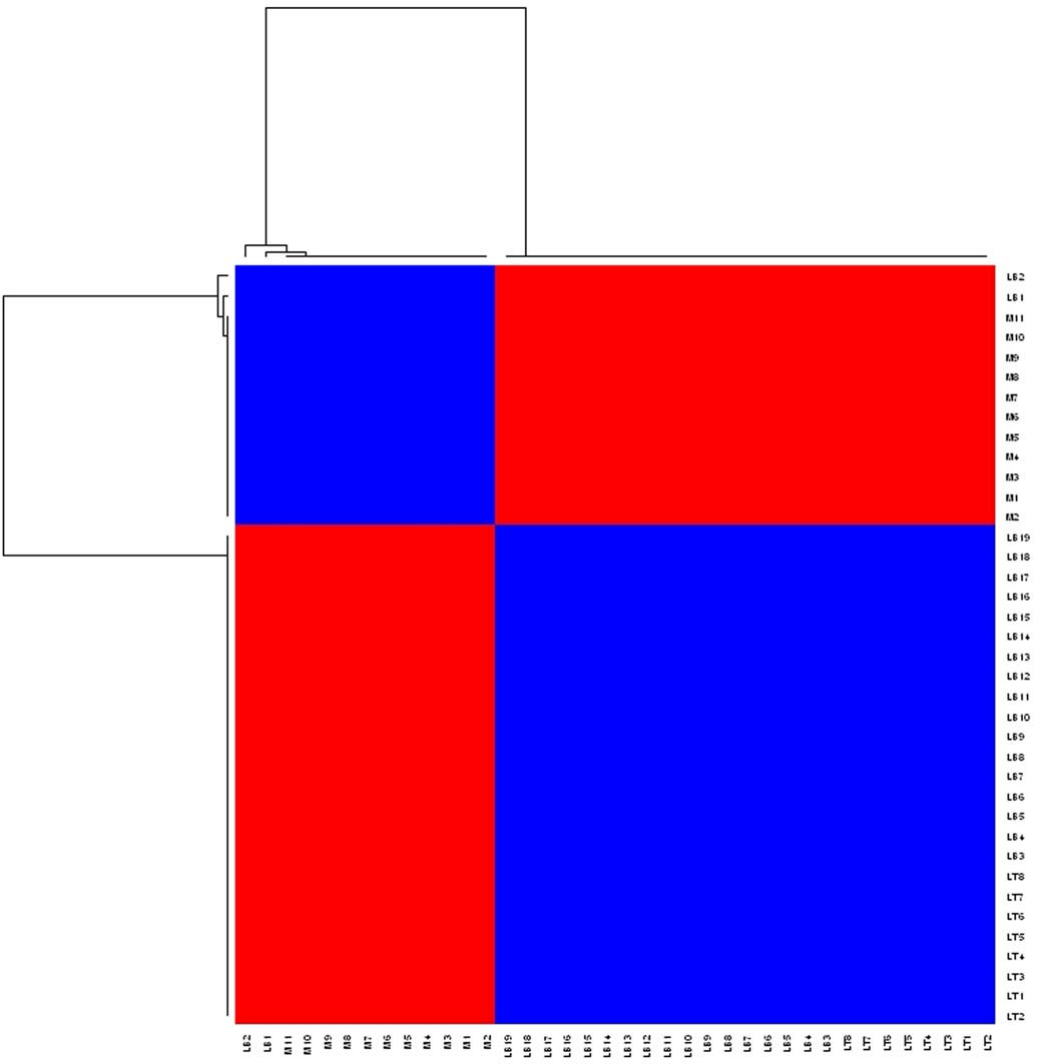
Heat map of re-ordered consensus matrix, *k* = 2.

**Figure 5:**
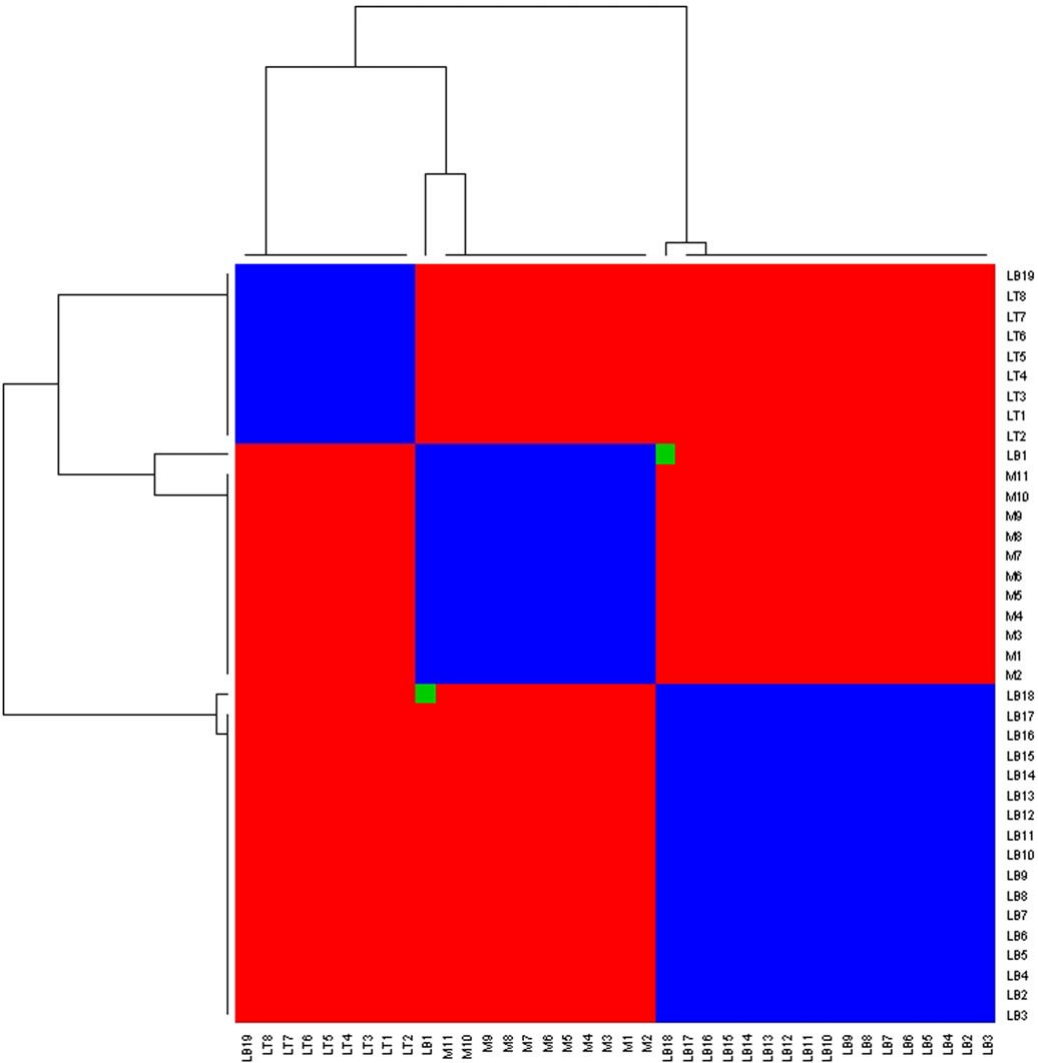
Heat map of re-ordered consensus matrix, *k* = 3.

Returning to the leukemia example, we applied factorizations of ranks *k* = 2,3,4,5 based on Equation 1 for 200 runs each. Figure 3 plots *ρ* versus *k* where *ρ* starts falling off sharply after *k* = 2. Figures 4 and 5 show heat maps of the re-ordered consensus matrices based on HC for *k* = 2,3 (for details see [38]). The homogeneity of coloring seen in these graphs indicate the presence of 2 and 3 clusters of samples, delineating the ALL and AML classes as well as the B and T subtypes within the ALL class. Again, “L” and “M” denote, respectively, an ALL and an AML sample, while “B” and “T” denote the two ALL subtypes. In each case, two samples are misclassified by the method.

Other approaches to handling the information across multiple runs are also possible [40],[42]. For example, Kim and Tidor [40] plotted the root-mean-squared error (RMSE) between the original and NMF-reconstructed data as a function of the rank *k* and used it to choose the appropriate value of *k*. While the use of RMSE is appropriate when the factorization is based on Euclidean distance, it is important to note that other cost functions require the error to be modified accordingly. For a given rank *k* factorization, they also demonstrate reproducibility of the metagenes across multiple runs in terms of correlation between pairs. Furthermore, they show that NMF is robust to the addition of noise to the original data based on the mean correlation of the corresponding metagenes across multiple runs, suggesting its potential usefulness as a noise-reduction filter.

#### Implementation of the NMF Algorithm

The implementation of the steps in the model selection procedure outlined above is computationally very intensive for any real large-scale biological dataset. However, the stochastic nature of the algorithm enables each of these steps to be run independently and simultaneously. These steps can be repeated for multiple random initial conditions for *W* and *H*, and the information from the independent runs combined via consensus clustering. Thus, the NMF algorithm lends itself easily to a parallel implementation that would greatly increase speed and efficiency. Devarajan and Wang [71] outlined such a parallel implementation of this algorithm on a Message-Passing Interface/C++ platform (http://www-unix.mcs.anl.gov/mpi/mpich2/) using high-performance computing clusters.

Recently, there have also been other efforts to optimize the implementation of this algorithm. Okun and Priisalu [54],[72] have reported faster convergence of the algorithm when feature scaling is applied to the original *p*×*n* data matrix *V*, i.e., each of the *p* rows of *V* is normalized to have values between 0 and 1. Their results indicate an increase in speed of at least 11 times in the convergence of iterations due to such normalization, depending on the number of latent factors *k* used in the factorization.

#### Identifying Hierarchical Structure

It is also possible to have overlapping metagenes, i.e., genes with non-zero coefficients can appear in multiple metagenes, indicating the role of a single or a group of genes in multiple pathways or processes. The stochastic nature of the algorithm can also be exploited towards identifying the involvement of such a group of genes. This is in contrast to most standard approaches for clustering large-scale biological data, with a few exceptions [51]. These standard methods provide only a single solution determined by the dominant or overall structure in the data where genes and samples are assigned to only one cluster, thus limiting the possibility of identifying overlapping expression patterns [38].

One of the attractive features of NMF is that, unlike HC, it does not force a hierarchy into the data structure but identifies one when it is present. By specifying the desired rank of the factorization, one can uncover substructures in the data in an ordered sequential manner. Brunet et al. [38] and Devarajan [37] have demonstrated the ability of NMF to identify hierarchical and nested sub-structures using cancer microarray data. Brunet et al. [38] noted that NMF has higher resolution than HC and is more stable than SOM as well as being more robust and less sensitive to a priori selection of genes. They also show that NMF always converges towards a fixed attractor irrespective of random initial conditions in comparison with a similar stochastic method like SOM.

For instance, in applying HC to the leukemia data to cluster the tissue samples, they note that the tree structure produced by HC depended very much on the choice of the linkage metric used in constructing the dendogram. Furthermore, they observed that the performance of HC varied depending on the number of input genes used in the clustering. Similarly, the authors applied SOM to this data and observed that for *k* = 2, the clustering was unstable and depended on the random initial conditions, while for *k* = 3, the method was unable to recover the three tumor types (for details see [38]). In sharp contrast, NMF with rank *k* = 2 was able to consistently recover the distinction between the ALL and AML types. This is reflected in the homogeneity in coloring of a heat map of the re-ordered consensus matrix shown in Figure 4 and the high cophenetic correlation coefficient for this case (see Figure 3). Likewise, a rank *k* = 3 factorization was able to consistently recover the distinction between the ALL-B and -T subtypes as seen in Figure 5.

## Some Limitations

A review of this widely applicable method would not be complete without a discussion of its limitations. As noted earlier, NMF is an algorithmically more complex method to implement, and convergence can be slow. This is further compounded by the stochastic nature of the algorithm despite its obvious advantages as outlined in the previous section. The standard NMF formulation does not incorporate statistical dependencies between the metagenes or metagene expression patterns, nor does it identify any structural relationships between them. Also, the parts-based representation may be holistic, rather than local, depending on the type and nature of the data being studied [19], [23]–[25]. The nonnegativity constraints that are critical to such a representation may not be sufficient to achieve sparseness in some situations. Then, one would have to explicitly enforce sparseness by the addition of appropriate penalty terms to the cost function being used in the decomposition. In such cases, it is also possible that a parts-based, local representation may require fully hierarchical models with multiple levels of hidden variables rather than the single level used in this approach [1]. The issue of normalization of the observed data prior to NMF analysis is an important problem and one that has not been systematically studied. Some normalization methods have been suggested in the literature [50],[54],[72], but it would be useful to assess and compare the impact of different methods on the decomposition itself.

## Discussion

In the NMF formulation, both the metagenes and the metagene expression patterns are nonnegative and sparse, and this is a key requirement for a parts-based local representation. Sparseness has been demonstrated to capture context-dependent biological patterns based on only a small subset of genes or samples. The alternating feature of the algorithm as defined by the multiplicative update rules facilitates simultaneous inference and learning [1],[2],[37] from the metagenes and metagene expression patterns. The stochastic nature of the NMF algorithm provides a means to evaluate its sensitivity towards random initial conditions as well as in assessing whether a given rank *k* provides a biologically meaningful decomposition of the data. Furthermore, this feature has been successfully utilized in identifying hierarchical structure within the data and in the implementation of parallel algorithms to increase speed and efficiency. Perhaps one of the most useful applications of NMF is in metagene projection, for cross-platform, cross-species analyses and interpretation of large-scale biological data. This approach not only reduces noise and technological variations in the data but can also incorporate prior knowledge in characterizing new datasets.

In the previous section, we noted that NMF does not account for dependencies in the metagenes or metagene expression patterns. However, in certain applications, it may be relevant to explicitly include or exclude dependencies in these hidden variables. For instance, independent component analysis (ICA) [73],[74] is an approach that produces statistically independent non-Gaussian components. There has been some work extending ICA to include nonnegativity constraints [75]–[77]. It would be potentially useful to extend this to include other dependent structures within these hidden variables.

In summary, NMF is an emerging new paradigm for large-scale biological data analysis and interpretation. It offers tremendous potential for applicability in a wide variety of computational biology problems as evidenced by the recent surge in literature. The relevance of this approach for text mining and document clustering also makes it a potentially indispensable tool in biomedical informatics. Last but not least, its applicability is not just limited to biological problems but encompasses diverse areas such as image and sound processing, text mining, and information retrieval.
